# Correlation Between Reduction Quality of Femoral Neck Fracture and Femoral Head Necrosis Based on Biomechanics

**DOI:** 10.1111/os.12458

**Published:** 2019-04-26

**Authors:** Ying Wang, Jian‐xiong Ma, Tao Yin, Zhe Han, Shuang‐shuang Cui, Zhi‐peng Liu, Xin‐long Ma

**Affiliations:** ^1^ Institute of Biomedical Engineering Chinese Academy of Medical Sciences and Peking Union Medical College Tianjin China; ^2^ Digital Orthopaedic Laboratories of the Orthopaedic Institute Tianjin Hospital Tianjin China

**Keywords:** Femoral head necrosis, Femoral neck fracture, Finite element analysis, Gait, Reduction quality

## Abstract

**Objective:**

To investigate the biomechanical effects of reduction quality on patients after femoral neck fracture internal fixation.

**Methods:**

The data of individual patients with femoral neck fractures were reviewed. Data for patients with simple unilateral femoral neck fractures whose reduction quality was evaluated as good by hip X‐ray films after internal fixation were collected from January 2013 to January 2017. The CT data of the patients was used to reconstruct 3D models of the femur and the screw. The spatial displacement after the operation of femoral neck fracture was measured, which included the displacement of the deepest portion of the femoral head fovea, the displacement of the center of the femoral head, and the rotational angle. The cases were followed up by telephone consultation and clinical review to determine whether the osteonecrosis of the femoral head occurred. Follow‐up time should be more than 18 months after surgery. The cases were grouped according to the results into an osteonecrosis of the femoral head group and a non‐osteonecrosis of the femoral head group. Finally, the differences in postoperative spatial displacement between the two groups were compared and analyzed. In addition, a mechanical analysis of femoral force during gait was performed *via* finite element analysis.

**Results:**

Data for 241 patients with femoral neck fractures who were treated with closed reduction and internal fixation were collected. 3D measurement showed the average displacement value, including the center of the femoral head (5.90 ± 3.4 mm), the deepest portion of the femoral head fovea (9.32 ± 4.8 mm), and the rotational angle (16.1° ± 9.4°). After telephone consultation and clinical review, osteonecrosis of the femoral head was diagnosed in 28 (11.62%) of the patients. In the osteonecrosis of the femoral head (ONFH) group, the displacement of the deepest portion of the femoral head fovea was 10.92 ± 9.18 mm; the displacement was 8.86 ± 6.29 mm in the non‐ONFH group. The displacement of the center of the femoral head in the ONFH group was 7.575 ± 5.69 mm and 5.31 ± 4.05 mm in non‐ONFH group. The rotational angle was 20.11° ± 10.27° in the ONFH group and 14.19° ± 11.09° in the non‐ONFH group. The statistical analysis showed that the postoperative spatial displacements, including the displacement of the deepest portion of the femoral head fovea, the displacement of the center of the femoral head, and the rotational angle between the two groups, had statistical differences. Finite element analysis showed that as the spatial displacement increased, the stress, the displacement, and the equivalent strain of the proximal femur also increased.

**Conclusion:**

Poor reduction quality after femoral neck fracture is a risk factor for re‐fracture and femoral head necrosis, and the measurement method of this study can be used to predict the occurrence of femoral head necrosis early after femoral neck fracture.

## Introduction

Femoral neck fractures are a common type of traumatic injury accounting for approximately 54% of hip fractures^1^ and 3.58% of body fractures^2^. Femoral neck fractures mainly occur in elderly individuals after falls and are injuries associated with indirect violence and low energy^3^. With the advent of an aging society, the incidence of femoral neck fractures is on the rise, making femoral neck fracture a non‐negligible problem in China, as well as the rest of Asia and the world^1^, ^4^.

Owing to the anatomical structure and biomechanical characteristics of the femoral neck, complications such as nonunion and osteonecrosis of the femoral head (ONFH) can easily occur after fracture, which cause high disability and mortality^5^. The treatment of femoral neck fractures continues to progress, but the incidence of femoral head necrosis after fractures still fluctuates between 8.1% and 37.2%^6^. Therefore, femoral neck fractures have been referred to as “unresolved fractures”^7^. At the same time, the high incidence of traffic accidents makes femoral head necrosis more relevant to the health of adolescents. As reported, the incidence of ONFH after fractures in adolescents is obviously higher than that in the elderly^8^.

After the internal fixation of femoral neck fracture, the stress is mainly concentrated on the fracture. Thus, only with complete anatomical reduction of the fracture can shearing, torsion, and bending be resisted^9^. Zlowodzki^10^ investigated 298 orthopaedic surgeons’ understanding of prognostic factors related to femoral neck fractures, and the quality of fracture reduction was considered to be the most important factor. Palm^11^ conducted a follow‐up study on patients with femoral neck fractures treated with reduction and internal fixation and found that the quality of fracture reduction has an obvious correlation with the incidence of complications such as ONFH. He also reported that poor reduction of fractures can lead to pain, nonunion of fractures and even secondary surgery. Beris’s research further confirmed that the primary factor affecting prognosis is the quality of fracture reduction. In addition, he reported that the earlier a high‐quality reduction is performed, the higher the healing rate of the fracture and the lower the rate of ONFH^12^.

The method for accurately assessing the quality of reduction after femoral neck fractures has important clinical significance. Garden classification is the most common method for assessing the quality of fracture reduction, which is based on X‐rays. However, the fact that X‐rays are 2D images generated from a projected 3D image results in image overlap, occlusions, and distortions, which limit the evaluation of fracture displacement and rotation^13^. A 3D reconstruction technique based on a CT workstation has also been proposed for the evaluation of postoperative reduction quality of femoral fractures. However, this method has been shown to be unable to completely separate the femoral part on its own, and the overlapping occlusion of the anatomy at the hip joint makes it difficult for the clinician to correctly judge the quality of the reduction^14^. In this study, the reduction quality of the femoral neck fracture was evaluated using the method of healthy femur and postoperative femoral matching. In addition, the residual displacement of poor reduction quality was quantified, which led us to reassess the reliability of evaluations based on X‐ray. At the same time, a method for accurately evaluating the quality of postoperative reduction of femoral neck fractures was proposed.

Recently, it was acknowledged that the occurrence of femoral head necrosis is associated with the postoperative reduction quality of femoral neck fractures^15^, ^16^. Femoral head necrosis was first thought to be caused by avascular necrosis. However, with further research on the femoral head, many researchers have found that necrosis may also occur in areas with good blood supply. Besides, it has been reported that the femoral head has a high metabolic reaction in the early stage of collapse and after collapse. Finally, it was found that the distribution of femoral head necrosis was not consistent with the distribution of blood supply. However, the stress concentration area of the femoral head in daily life is the area of femoral head necrosis and collapse. In addition, there are many animal studies confirming the theory of mechanical factors of femoral head necrosis.

However, there have been few reports on the effects of poor reduction quality on the biomechanical mechanisms of the femur. Our team studied the stress, displacement, and equivalent strain of the femur under normal anteversion. With changes in the anteversion angle (whether increases or decreases), the effective stress and displacement of the proximal femur gradually increase. Therefore, residual femoral neck displacement will cause greater adverse stress and displacement at the proximal end of the femur.

The purpose of this study was: (i) to evaluate the quality of femoral head reduction using CT data from patients with clinical femoral neck fractures and to verify the limitations of fracture reduction during 2D image guidance; (ii) to simulate the effect of poor reduction quality of femoral neck fracture on the femoral force during walking and to explore its biomechanical mechanism using the finite element analysis (FEA) method; and (iii) to verify the correlation between the quality of poor reduction of femoral neck fracture and the occurrence of femoral head necrosis by follow‐up.

## Materials and Methods

### *Database of Patients with Femoral Neck Fractures*

The data of individual patients with femoral neck fractures in Tianjin Hospital from January 2013 to January 2017 were reviewed. The patients who met the inclusion criteria were screened to establish a database of patients with femoral neck fractures. Inclusion criteria followed the PICOS principle: (i) Participant: simple unilateral femoral neck fractures in patients aged 20–75 years; (ii) Intervention: after the internal fixation, the hip X‐ray film showed good quality of the reduction, and CT imaging was performed after operation. (iii) follow‐up of more than 18 months; (iv) Comparison: the spatial displacement after the operation of femoral neck fracture was compared between the ONFH group and the non‐ONFH group; (v) Outcome: the spatial displacement of the ONFH group should be greater than that of the non‐ONFH group; and (vi) Study design: retrospective study. Exclusion criteria: (i) multiple fractures; (ii) pathological fractures, bilateral femoral neck fractures, and old fractures; (iii) bone tumor malformations; (iv) postoperative infection or steroid‐induced avascular necrosis of the femoral head; (v) no postoperative CT data; and (vi) patients and their families did not sign informed consent.

### *Reconstruction and Registration Model*

The CT data of the patients were imported into Simpleware version 7.0 (Simpleware, UK) in DICOM format, which generated 3D models of the bilateral proximal femurs. A mirror model of the normal side was produced by the mirror function of the software. Then, the fractured femur was superimposed on the mirror model. If there was an incomplete reduction after femoral neck fracture, the femoral head would have a certain offset after the bilateral femoral neck and femoral shaft were matched.

### *Three‐dimensional Measurement*

The femur is the main load‐bearing organ and bears almost all the weight of the human body. Any displacement will affect the biomechanical transmission of the femur, causing complications after femoral neck fracture internal fixation. Therefore, the spatial displacement after the operation of femoral neck fracture was measured, which included the displacement of the deepest portion of the femoral head fovea, the displacement of the center of the femoral head, and the rotational angle.

#### *Displacement of the Deepest Portion of the Femoral Head Fovea*

The fovea of the femoral head is a typical anatomical point on the femoral head, which is easy to identify. The displacement of the deepest portion of the femoral head fovea was the distance between the deepest points of the fovea of the femoral heads matched. It represents the extent of the medial displacement of the femoral head.

#### *Displacement of the Center of the Femoral Head*

The femoral head is a hemispherical structure. Thus, in the present study the femoral head was fitted as a ball using software, and the center of the ball was the center of the femoral head. The displacement of the center of the femoral head was the distance between the centers of the femoral heads matched. It represents the overall degree of displacement of the femoral head.

#### *Rotational Angle of the Femoral Head*

In the fracture model, a line was drawn through the center of the femoral head and the deepest point of the femoral head fovea. The same line was then drawn on a mirror model. An angle was formed by the two lines and was recorded to describe the rotation of the femoral head postoperatively, which was the rotational angle. It quantifies femoral head deflection from an angle.

### *Follow‐Up of Cases*

Moreover, the cases were followed up to determine whether ONFH occurred and were then grouped according to the results into an ONFH group and a non‐ONFH group. Finally, the differences in postoperative spatial displacement between the two groups were compared and analyzed.

### *Finite Element Analysis*

Random sampling was conducted among the patients in the database, and 30 cases were selected for biomechanical finite element analysis. The 3D models of the femoral region and the internal fixation screw for the femoral neck fractures of the selected patients were assigned in the Simpleware software. Material assignment based on the gray value in the CT data was as follows:

(1) $$\left\{\begin{array}{l} \text{Density}=131+1.067\times \mathrm{HU}\\ {\text{Young}}^{'}s\;\text{modulus}=-331+4.56\times \mathrm{rho}\\ \text{Poisson ratio} \end{array}\right..$$

The screw had a density of 4500 kg/m^3^; the Young’s modulus was 108 000 MPa; the Poisson ratio was 0.3^17^. Then, the internal fixation and femoral models were meshed and the finite element models were established. Because the focus of this study was not related to the internal fixation thread, the thread details were ignored to simplify the model, and the contact surface between the cannulated screw model and the proximal femoral fracture model was set to no friction. Finally, mechanical loading analysis was performed using the Abaqus version 6.14 software (Dassault Simulia, USA). The gait was divided into four periods, including: a two‐legged standing period, when the femur had to bear a load of 1/3 of the weight; a one‐legged standing period, when the femur had to bear a load of 2.4 times the weight; the heel‐off period, when the femur had to bear a load of 4 times the weight; and the toe‐off period, when the femur had to bear a load of 7 times the weight^18^. The stress, strain, and displacement distribution on the finite element model of the proximal femur after internal fixation during the gait period were observed and analyzed.

### *Statistical Method*

SPSS version 21 (IBM, USA) was applied, and the measurement data are represented as (mean ± standard deviation). Before analyzing all the data, the Shapiro–Wilk test and the Bartlett test were used to judge whether the data were normally distributed and whether the variance was homogeneous. When the data were considered to present a normal distribution and homogeneity of variance, the independent sample *t*‐test was used; otherwise, the rank sum was adopted. *P* < 0.05 was considered to be statistically significant.

## Results

### *Database of Patients with Femoral Neck Fractures*

A total of 241 patients with femoral neck fractures who were treated with closed reduction and internal fixation were collected at Tianjin Hospital from January 2013 to January 2017. This cohort included 101 men and 140 women, with an average age of 53.46 years.

### *Measurement and Follow‐up Results*

**Figure 1:**
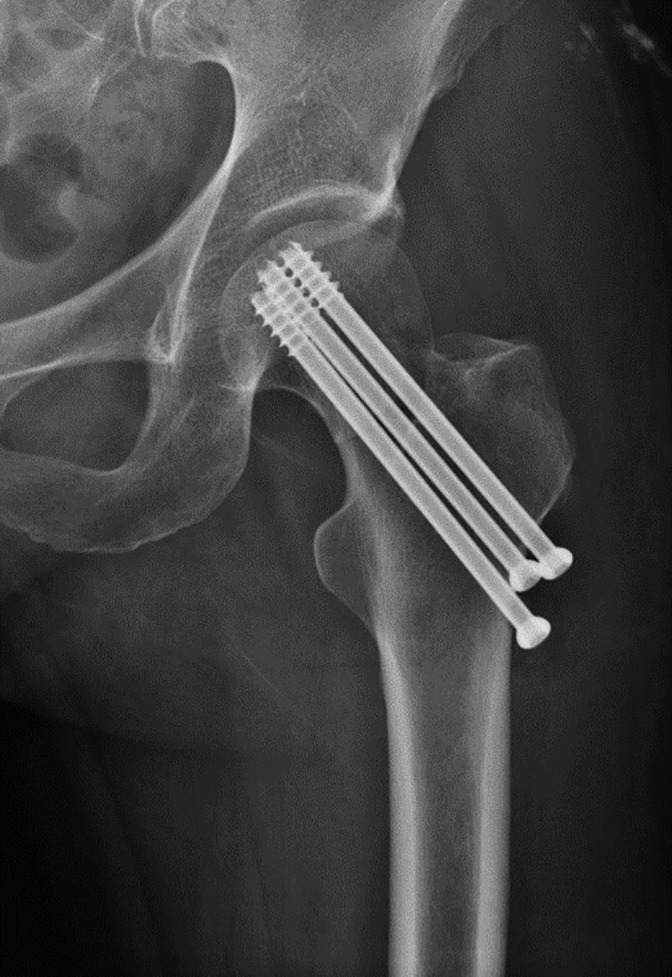
Antero–posterior postoperative radiograph of a right femoral neck fracture in a 58‐year‐old woman.

**Figure 2:**
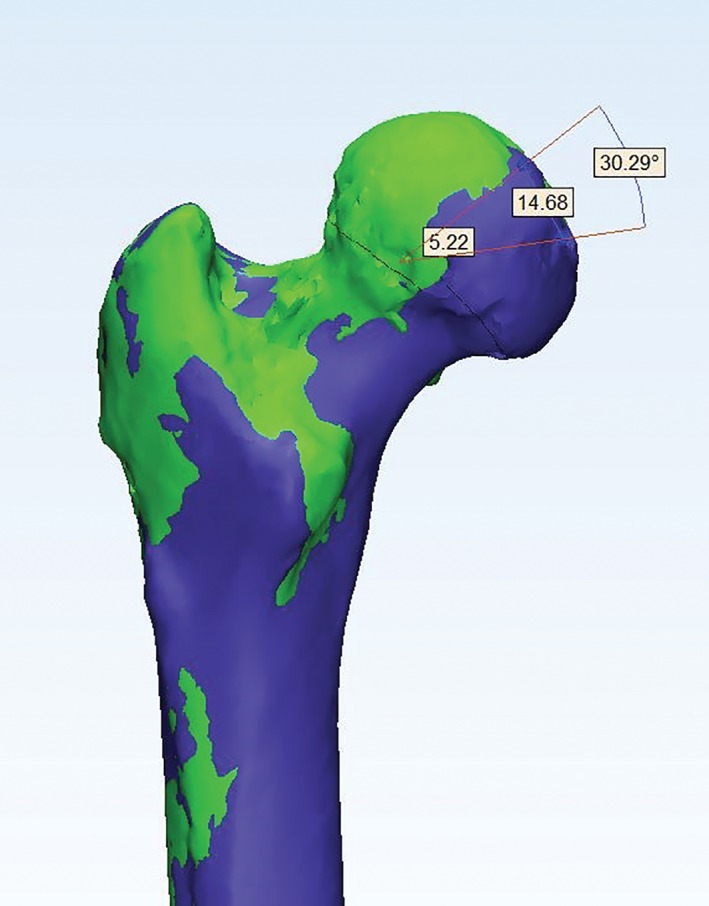
Front views of the proximal femoral 3D model and mirror model and the residual displacement of the femoral head.

In 241 patients with internal femoral neck fractures (Fig. 1), the average displacement of the center of the femoral head was 5.90 ± 3.4 mm; the average displacement of the deepest portion of the femoral head fovea was 9.32 ± 4.8 mm; and the rotational angle was 16.1° ± 9.4° (Fig. 2). After the telephone consultation and clinical review, osteonecrosis of the femoral head was diagnosed in 11.62%, or 28, of the patients. According to sex, 17 of these patients were men and 11 were women. Their average age was 52.9 ± 8.1 years and the mean time to necrosis was 1.4 ± 0.7 years after surgery. Thus, the ONFH group had 28 cases and the non‐ONFH group had 213 cases. According to the statistical analysis, the postoperative spatial displacements, including the displacement of the deepest portion of the femoral head fovea, the displacement of the center of the femoral head, and the rotational angle between the two groups, had statistical differences (Table 1).

**Table 1:** The associations between residual displacement and ONFH after femoral neck fracture (mean ± standard deviation)

Groups	Displacement of the deepest portion of the femoral head fovea (mm)	Displacement of the center of the femoral head (mm)	Rotational angle (°)
ONFH (*n* = 28)	10.92 ± 9.18	7.575 ± 5.69	20.11 ± 10.27
Non‐ONFH (*n* = 213)	8.86 ± 6.29	5.31 ± 4.05	14.19 ± 11.09
*P*	0.026	0.005	0.005

*P* < 0.05 was defined as indicating a statistically significant difference. ONFH, osteonecrosis of the femoral head.

### *Finite Element Analysis Result*

**Figure 3:**
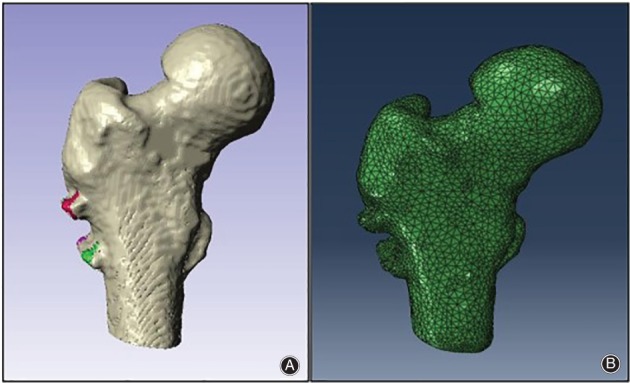
Reconstruction and mesh model of femoral neck fracture: (A) 3D model of the proximal femur after cannulated screw internal fixation; (B) 3D mesh model of the proximal femur after cannulated screw internal fixation.

Thirty cases of femoral neck fracture internal fixation were randomly selected for finite element analysis (Fig. 3); 12 of these patients were men and 18 were women. Their average age was 54.4 ± 10.5 years. The average displacement of the center of the femoral head was 5.32 ± 3.0 mm; the average displacement of the deepest portion of the femoral head fovea was 9.22 ± 4.4 mm; and the rotational angle was 19.73° ± 11.6°. The stress–strain and displacement changes in the four state periods during the gait of the patients were analyzed; these periods were the two‐legged standing period, the one‐legged standing period, the heel‐off period and the toe‐off period.

**Figure 4:**
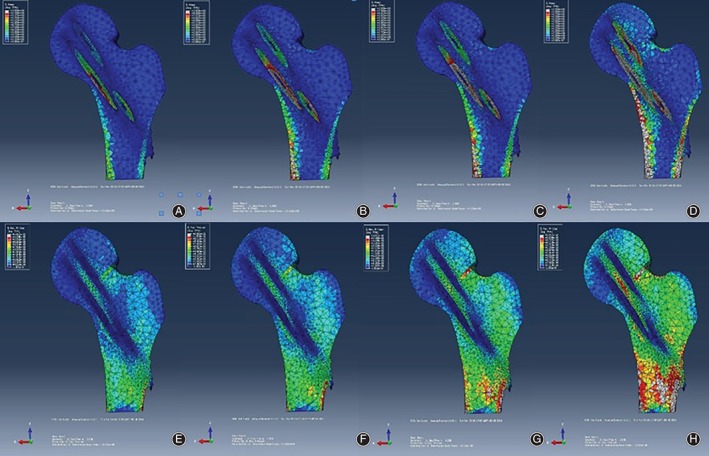
Stress–strain distribution of a patient during gait: (A) indicates the stress distribution of the two‐legged standing period; (B) indicates the stress distribution of the one‐legged standing period; (C) indicates the stress distribution of the heel‐off period; (D) indicates the stress distribution of the toe‐off period; (E) indicates the strain distribution of the two‐legged standing period; (F) indicates the strain distribution of the one‐legged standing period; (G) indicates the strain distribution of the heel‐off period; and (H) indicates the strain distribution of the toe‐off period.

Finite element analysis was solved, and the values obtained from the solution are provided in Table 2. The results of FEA showed that the stress–strain was mainly concentrated in the upper and lower cortices and lateral cortex of the femoral neck during gait (Fig. 4). The stress, displacement, and equivalent strain of the proximal femur were maximized during the toe‐off period. In any period of gait, as the spatial displacement of the femoral head increased, the stress, displacement, and equivalent strain of the proximal femur also increased.

**Table 2:** Mechanical properties of the proximal femur in each state of gait (mean ± standard deviation)

State period of gait	Stress peak (MPa)	Equivalent strain (uε)	Maximum displacement (mm)
Two‐legged standing period	22.09 ± 6.2	283.77 ± 103.86	0.049 ± 0.013
One‐legged standing period	159.04 ± 44.69	2043.13 ± 747.82	0.36 ± 0.92
Heel‐off period	265.07 ± 74.48	3405.22 ± 1246.37	0.59 ± 0.15
Toe‐off period	463.88 ± 130.33	5959.14 ± 2181.15	1.04 ± 0.27

## Discussion

### *Accuracy of Measurement Method*

All cases included in this study involved unilateral femoral neck fractures. The adopted method was measurement of the residual displacement of the affected femoral neck based on the contralateral femoral neck. Our team also measured and compared the normal bilateral femoral neck in Chinese individuals (Table 3). The results showed that there was no significant difference between the left and right sides (*P* > 0.05). Therefore, the method based on the contralateral femoral neck in this study was feasible.

**Table 3:** Parameter values for the proximal femur (in mm); mean ± standard deviation

Parameters	Left	Right
Diameter of the femoral head	45.09 ± 3.74	46.32 ± 4.23
Height of the femoral head	52.99 ± 5.81	54.23 ± 5.09
Eccentricity	39.71 ± 4.95	40.11 ± 5.30
Central diameter of the femoral neck	37.21 ± 3.28	36.42 ± 3.73
Collodiaphysial angle	128.24 ± 6.12	127.52 ± 6.32
Length of femoral neck	47.55 ± 3.68	45.67 ± 4.94
Intertrochanteric distance	52.14 ± 4.87	53.75 ± 5.12
Inner diameter of the medullary cavity	26.83 ± 4.12	25.58 ± 4.79

### *Follow‐up Results*

Femoral neck fractures have been referred to as “unresolved fractures”^7^ because of the complications such as nonunion of the fracture and ONFH. Although the development of internal fixation techniques improves the rate of fracture nonunion, the incidence of ONFH continues to fluctuate between 8.1% and 40% as reported^19^, ^20^. In this study, the necrosis rate was 10%, which may have some bias due to the bias of the fracture type. Some studies have reported that the incidence of ONFH in displaced Garden III and IV fractures is significantly higher than in nondisplaced fractures^21^, ^22^. In this study, the Garden I type of femoral neck fracture accounted for 28.6% of the total cases. Among the cases of ONFH, 1 case involved a Garden I type fracture, 5 cases involved Garden II type fractures, 12 cases involved Garden III type fractures, and 10 cases were classified as Garden IV. Regarding the type of fracture, similar to the previously reported results, displaced femoral neck fractures were more prone to ONFH than nondisplaced fractures.

The statistical analysis of the follow‐up data of some cases showed that no significant differences existed between the groups regarding age, sex, fracture laterality, the mechanism of injury, or implant configuration. However, there is controversy about the correlation between the time interval from injury to surgery and the occurrence of ONFH. Jain^23^ reported that the rate of ONFH in patients undergoing internal fixation within 6 h was lower than that in patients undergoing internal fixation after 12 h. However, Razik^24^ considered the time from injury to surgery to have no significant effect on the occurrence of ONFH. Although our research results were consistent with Razik’s viewpoint, early surgery can be recommended because early surgery can reopen twisted, deformed, and stenotic blood vessels, and the pressure of the joint capsule is conducive to the recovery of the blood supply to the femoral head.

### *Finite Element Results*

The finite element results showed that the stress distribution at the proximal femur also changed significantly with the spatial displacement and angle of the femoral head. The stress concentration was gradually transferred from the femur to the upper part of the head–neck junction. Then, the direction of the force of the femoral head would no longer coincide with the trabecular orientation, resulting in changes in the distribution of the ultrastructural trabecular bone^25^. In this situation, the trabecular bone at the femoral head is subjected not only to axial stress but also to shear stress, which is detrimental to the stability of the trabecular structure. Our research also showed that in different state periods of gait, as the spatial angle and displacement of the femoral head increased, the maximum strain load at the proximal femur increased, with the greatest load being observed in the toe‐off period; the maximum value was 9464.49 uε, and the average value reached 5591.13 uε. Our team^26^ has shown that when the load exceeds 3000 uε, it will cause accumulation of micro‐damage of bone tissue, and when the load exceeds 5000 uε, there will be a risk of fracture. Therefore, in our research, bones would be reconstructed or remodeled if such adverse stimuli persist, resulting in changes in the internal distribution of trabecular bone but poor structural stability. When the increase in bone mass cannot compensate for this long‐term high stress stimulus, the reconstruction of the bone unit will be blocked. At this time, the micro‐fracture of the trabecular bone cannot be repaired, which will cause the surface of the femoral head to collapse and eventually undergo necrosis^27^. The follow‐up results of this study showed that the spatial displacement and angle of the ONFH group were significantly higher than those of the non‐ONFH group. The 30 patients randomized in this study included 7 patients with necrosis. The mechanical analysis showed that the stress, equivalent strain, and displacement of the necrotic cases were greater than those of the nonnecrotic cases in the gait, but the statistical results showed that only the displacement peaks presented significant differences.

### *Limitation of the Study*

There were some limitations of our research that should be noted, as follows: (i) the finite element analysis did not include the effects of soft tissues around the hip joints, such as ligaments and muscles; and (ii) this study was based on the traditional theory and method of continuum mechanics. On the basis of in‐depth analysis of bone structure, the bone is abstracted into a modeled engineering material, and the linear elastic mechanical model of the proximal femur is assumed. However, the bone is, in fact, an anisotropic material^28^.

This report is the first study to describe the biomechanical effects of a poor quality of femoral neck fracture reduction on gait. Moreover, the results of this study confirmed the unreliability of the standard widely used in clinical for evaluating the reduction quality of femoral neck fractures based on X‐ray and provide clinicians with research direction in the treatment of femoral neck fractures. Meanwhile, this study outlines a method for early prediction of femoral head necrosis after femoral neck fracture, which can facilitate early intervention by doctors.

## References

[1] Thorngren KG , Hommel A , Norrman PO , Thorngren J , Wingstrand H . Epidemiology of femoral neck fractures. Injury, 2002, 33: 1–7. 12423584 10.1016/s0020-1383(02)00324-8

[2] Tian FM , Zhang L , Zhao HY , Liang CY , Zhang N , Song HP . An increase in the incidence of hip fractures in Tangshan, China. Osteoporos Int, 2014, 25: 1321–1325. 24562838 10.1007/s00198-013-2600-6

[3] Orive M , Aguirre U , Garcla GS , *et al* Changes in health‐related quality of life and activities of daily living after hip fracture because of a fall in elderly patients: a prospective cohort study. Int J Clin Pract, 2015, 69: 491–500. 25721490 10.1111/ijcp.12527

[4] Qian JG , Tang X . Examination of femoral‐neck structure using finite element model and bone mineral density using dual‐energy x‐ray absorptiometry. Clin Biomech (Bristol, Avon), 2009, 24: 47–52. 10.1016/j.clinbiomech.2008.09.007 18980785

[5] BachIller FG , Caballer AP , Portal LF . Avascular necrosis of the femoral head after femoral neck fracture. Clin Orthop Relat Res, 2002, 399: 87–109. 10.1097/00003086-200206000-00012 12011698

[6] Sun YQ , Chen LL , Liu YH , *et al* Current status of research on femoral head necrosis after internal fixation of femoral neck fracture. Chin J Tissue Eng Res, 2017, 21: 3095–3101.

[7] Li MN , Cole PA . Anatomical considerations in adult femoral neck fractures: how anatomy influences the treatment issues. Injury, 2015, 46: 453–458. 25549821 10.1016/j.injury.2014.11.017

[8] Slobogean GP , Stockton DJ , Zeng B . Femoral neck fractures in adults treated with internal fixation: a prospective multicenter Chinese cohort. J Am Acad Orthop Surg, 2017, 25: 297–303. 28248692 10.5435/JAAOS-D-15-00661

[9] Yoshimoto K , Nakashima Y , Nakamura A , *et al* Neck fracture of femoral stems with a sharp slot at the neck: biomechanical analysis. J Orthop Sci, 2015, 20: 881–887. 26209279 10.1007/s00776-015-0745-1

[10] Zlowodzki WB , Petrisor B . The value of washers in cannulated screw fixation of femoral neck fractures. J Trauma, 2005, 59: 969–975. 16374290 10.1097/01.ta.0000188130.99626.8c

[11] Palm H , Gosvig K , Krasheninnikoff M , Jacobsen S , Gebuhr P . A new measurement for posterior tilt predicts reoperation in undisplaced femoral neck fractures: 113 consecutive patients treated by internal fixation and followed for 1 year. Acta Orthop, 2009, 80: 303–307. 19634021 10.3109/17453670902967281 PMC2823202

[12] Beris AE , Kostopoulos VK . Non‐union of femoral neck fractures with osteonecrosis of the femoral head: treatment with combined free vascularized fibular grafting and subtrochanteric valgus osteotomy. Orthop Clin North Am, 2004, 35: 335–343. 15271541 10.1016/j.ocl.2004.02.008

[13] Avrahami D , Pajaczkowski JA . Femoral neck stress fracture in a female athlete:a case report. J Chiropr Med, 2012, 11: 273–279. 23843760 10.1016/j.jcm.2012.05.010 PMC3706699

[14] Casciaro ME , Ritacco LE , Milano F , Risk M , Craiem D . Angle estimation of human femora in a three‐dimensional virtual environment. Conference of the IEEE Engineering in Medicine and Biology. IEEE, 2010: 3946–3949. 10.1109/IEMBS.2010.5627701 21097090

[15] Ai ZS , Sun Y . Logistic regression analysis of factors associated with avascular necrosis of the femoral head following femoral neck fractures in middle‐aged and elderly patients. J Orthop Sci, 2013, 18: 271–276. 23114858 10.1007/s00776-012-0331-8

[16] Föger‐Samwald U , Vekszler G , Hörz‐Schuch E , *et al* Molecular mechanisms of osteoporotic hip fractures in elderly women. Exp Gerontol, 2016, 73: 49–58. 26608808 10.1016/j.exger.2015.11.012

[17] Wirtz DC , Pandorf T , Portheine F , *et al* Concept and development of an orthotropic FE model of the proximal femur. J Biomech, 2003, 36: 289–293. 12547369 10.1016/s0021-9290(02)00309-3

[18] Genda E , Li G . Normal hip joint contact pressure distribution in single‐leg standing‐effect of gender and anatomic parameters. J Biomech, 2001, 34: 895–905. 11410173 10.1016/s0021-9290(01)00041-0

[19] Lu YGL , Keller RB , Littenberg B , Wennberg JE . Outcomes after displaced fractures of the femoral neck. A meta‐analysis of one hundred and six published reports. J Bone Joint Surg Am, 1994, 76: 15–25. 8288658 10.2106/00004623-199401000-00003

[20] Min BW , Kim SJ . Avascular necrosis of the femoral head after osteosynthesis of femoral neck fracture. Orthopedics, 2011, 34: 349. 21598895 10.3928/01477447-20110317-13

[21] Lee KB , Howe TS , Chang HC . Cancellous screw fixation for femoral neck fractures: one hundred and sixteen patients. Ann Acad Med Singapore, 2004, 33: 248–251. 15098643

[22] Nikolopoulos KE , Papadakis SA , Kateros KT , *et al* Long‐term outcome of patients with avascular necrosis after internal fixation of femoral neck fractures. Injury, 2003, 34: 525–528. 12832180 10.1016/s0020-1383(02)00367-4

[23] Jain R , Koo M , Kreder HJ , Schemitsch EH , Davey JR , Mahomed NN . Comparison of early and delayed fixation of subcapital hip fractures in patients sixty years of age or less. J Bone Joint Surg Am, 2002, 84: 1605–1612. 12208917 10.2106/00004623-200209000-00013

[24] Razik F , Alexopoulos AS , El‐Osta B , *et al* Time to internal fixation of femoral neck fractures in patients under sixty years‐does this matter in the development of osteonecrosis of femoral head? Int Orthop, 2012, 36: 2127–2132. 22829122 10.1007/s00264-012-1619-1 PMC3460095

[25] Arlot ME , Burt‐Pichat B , Roux JP , Vashishth D , Bouxsein ML , Delmas PD . Microarchitecture influences microdamage accumulation in human vertebral trabecular bone. J Bone Miner Res, 2008, 23: 1613–1618. 18518771 10.1359/jbmr.080517 PMC3276353

[26] Ma XL , Fu X , Ma JX , *et al* Finite element study on spatial distribution and mechanical properties of cancellous bone from femoral head. J Med Biomech, 2010, 25: 465–470.

[27] Yu XZ . Finite element analysis of impact loads on the femur. Chin J Traumatol, 2007, 10: 44–48. 17229350

[28] Weng PW , Chen CH , Luo CA , *et al* The effects of tibia profile, distraction angle, and knee load on wedge instability and hinge fracture: a finite element study. Med Eng Phys, 2017, 42: 48–54. 28173978 10.1016/j.medengphy.2017.01.007

